# Whole-Exome Sequencing Identifies a Novel POLG Frameshift Variant in an Adult Patient Presenting with Progressive External Ophthalmoplegia and Mitochondrial DNA Depletion

**DOI:** 10.1155/2021/9969071

**Published:** 2021-11-05

**Authors:** Justin Kurtz, Joseph Americo Fernandes, Mahesh Mansukhani, William C. Copeland, Ali B. Naini

**Affiliations:** ^1^Division of Personalized Genomic Medicine, Department of Pathology and Cell Biology, Columbia University, 630 W. 168th Street, New York, NY 10032, USA; ^2^Department of Neurological Sciences, University of Nebraska Medical Center, Omaha, NE, USA; ^3^Mitochondrial DNA Replication Group, Genome Integrity and Structural Biology Laboratory, National Institute of Environmental Health Sciences (NIEHS), NIH, Research Triangle Park, NC 27709, USA; ^4^Department of Neurology, Columbia University, 630 W. 168th Street, New York, NY 10032, USA

## Abstract

Mitochondrial DNA (mtDNA) depletion syndromes are a group of autosomal recessive disorders associated with a spectrum of clinical diseases, which include progressive external ophthalmoplegia (PEO). They are caused by variants in nuclear DNA (nDNA) encoded genes, and the gene that encodes for mtDNA polymerase gamma (*POLG*) is commonly involved. A splice-site mutation in *POLG*, c.3104+3A > T, was previously identified in three families with findings of PEO, and studies demonstrated this variant to result in skipping of exon 19. Here, we report a 57-year-old female who presented with ophthalmoplegia, ptosis, muscle weakness, and exercise intolerance with a subsequent muscle biopsy demonstrating mitochondrial myopathy on histopathologic evaluation and multiple mtDNA deletions by southern blot analysis. Whole-exome sequencing identified the previously characterized c. 3104+3A > T splice-site mutation in compound heterozygosity with a novel frameshift variant, p.Gly23Serfs^*∗*^236 (c.67_88del). mtDNA copy number analysis performed on the patient's muscle showed mtDNA depletion, as expected in a patient with biallelic pathogenic mutations in *POLG*. This is the first reported case with *POLG* p.Gly23Serfs^*∗*^236, discovered in a patient presenting with features of PEO.

## 1. Introduction

Mitochondrial DNA (mtDNA) depletion syndromes (MDSs) are a diverse group of autosomal recessive disorders due to mutations in nuclear encoded genes [1]. They present with a broad phenotypic spectrum with disease manifesting in single or multiple organs, including the muscle, liver, brain, and kidney [2]. Based on the clinical phenotype, MDS can be classified into four categories: myopathic, encephalomyopathic, hepatocerebral, and neurogastrointestinal [3]. mtDNA depletion occurs when synthesis is insufficient to compensate for mtDNA turnover and segregation during cell division. It is caused by defects in genes involved in mtDNA replication or in genes involved in maintaining the pool of deoxyribonucleoside triphosphates (dNTPs) via the salvage pathway [2]. In addition to depletion, these defects may also lead to replication errors resulting in multiple mtDNA deletions [4].

The rate and timing of mtDNA synthesis is determined by the energy requirement of a cell [4]. mtDNA replication is independent of the cell cycle, but dNTPs are only formed by the de novo pathway during the S-phase of cell division [2]. Therefore, the salvage pathway is essential in maintaining a constant pool of dNTPs throughout the cell cycle, which is accomplished by synthesizing new dNTPs from preexisting deoxynucleosides [5]. Genes involved in the salvage pathway include TK2, DGUOK, SUCLA2, SUCLG1, RRM2B, and TYMP, and defects in any of these genes may result in mtDNA depletion due to an insufficient pool of dNTPs [2].

Autosomal recessive variants in the genes involved in mtDNA synthesis (*POLG*, *POLG2*, and *C10orf2*) are another cause of mtDNA depletion [2]. DNA polymerase gamma is the only known polymerase involved in the replication and maintenance of mtDNA in humans [6]. It is composed of two subunits encoded by nuclear genes (*POLG* and *POLG2*). *POLG* at chromosomal locus 15q25 encodes the catalytic subunit and contains a carboxy-terminus polymerase domain and an amino-terminus exonuclease domain [7]. Endogenous errors by the POLG protein during replication are the primary source for point mutations occurring in mtDNA [4, 8]. *POLG2* at chromosomal locus 17q24.1 encodes an accessory subunit [7]. The *C10orf2* gene encodes a DNA helicase (twinkle protein), which is essential for mtDNA replication [3].

POLG-related disorders exist on a spectrum with at least five major phenotypes, including (1) Alpers-Huttenlocher syndrome (AHS), (2) childhood myocerebrohepatopathy spectrum (MCHS), (3) myoclonic epilepsy myopathy sensory ataxia (MEMSA), (4) the ataxia neuropathy spectrum (ANS), and (5) progressive external ophthalmoplegia (PEO) with or without sensory ataxic neuropathy, dysarthria, and ophthalmoplegia (SANDO) [9]. There is no direct correlation between specific genotypic variants and the clinical or mtDNA phenotype; patients who are homozygous for identical *POLG* variants may have drastically different phenotypic findings [7, 10]. However, there are some data suggesting that the clinical phenotype correlates with the type of mtDNA alteration (deletions, depletion, or both) [7]. For example, the histopathologic findings in mitochondrial myopathies of ragged-red fibers and cytochrome c oxidase-negative fibers on muscle biopsy are a sign of increased mitochondria, and it correlates with the accumulation of mtDNA deletions in skeletal muscle [3, 11].

Here, we report a patient with late-onset PEO who was found to carry compound heterozygous variants in *POLG*. One allele carried the previously characterized *POLG* (NM_002693.3) : c.3104 + 3A > T splice-site alteration, which results in skipping of exon 19 [12, 13]. The other allele harbored a previously unreported frameshift variant, *POLG* (NM_002693.3) : c.67_88del (p.Gly23SerfsTer236). We performed additional studies to determine the structural/functional impact of this novel variant and its relationship to the patient's phenotype.

## 2. Case Presentation

A 57-year-old female was referred for evaluation of weakness and ptosis. She reported a history of being clumsy and less athletic than her peers as a child, such as not being able to run as fast. In her early 40s, she noticed difficulties climbing high steps, which progressed over time. She subsequently developed distal lower extremity weakness and started tripping. She reported an episode of becoming much weaker after a streptococcus infection, which slowly improved over several months. She was first diagnosed with ptosis around age 52 during a neurological evaluation. Her current symptoms include nonfatigable bilateral ptosis, vertical and horizontal ophthalmoplegia, bifacial weakness, weakness in the proximal and distal upper and lower extremities, worse in the lower extremities, and mild left-sided intention tremor. Electromyography demonstrated a myopathy with fibrillation potentials, worse in the lower extremities, without evidence of a peripheral neuropathy. Biceps muscle biopsy showed moderate fiber size variation with scattered atrophic and hypertrophic fibers, increased internal nuclei, fatty replacement of endomysial connective tissue on hematoxylin and eosin staining, “ragged red fibers” on Gomori Trichrome, focal muscle fibers with increased succinate dehydrogenase (SDH) activity (“ragged blue fibers”), and reduced or absent cytochrome c oxidase (COX) activity. Electron microscopy revealed increased mitochondria with abnormal cristae and crystalline inclusions. A diagnosis of mitochondrial myopathy was rendered.

## 3. Materials and Methods

### 3.1. Whole-Exome Sequencing

Whole-exome sequencing was performed on DNA extracted from the patient's muscle using the Agilent SureSelectXT Human All Exon V5+UTRs capture. Sequencing was performed on Illumina HiSeq2500 sequencing technology, and the data were analyzed for the presence of pathogenic mutations using the NextGENe software from Softgenetics and our in-house proprietary analytical pipeline. Sequencing variants were binned into groups based on their presence in databases of pathogenic variants, known disease-associated genes, and population frequency of the variant (above or below 1%). Variants in each of the bins were grouped based on whether they were “de novo” (not present in either parent), homozygous, compound heterozygous, or disruptive (stop-gain, frameshift, and canonical splice site). These were reviewed by two individuals using a “genetic” model (de novo variants for dominant conditions and homozygous or compound heterozygous variants for recessive conditions) as well as using a “candidate gene” approach based on clinical syndrome. GenBank sequence NM_002693.3 was used as the reference sequence for POLG.

### 3.2. Mitochondrial DNA Sequencing

Amplification of mtDNA was performed using four PCR primer pairs, and sequencing for variants in all 37 encoded genes was performed on Illumina MiSeq technology as previously described [14]. Variant detection was performed with NextGENe software from Softgenetics.

### 3.3. Mitochondrial DNA Deletion Analysis

DNA isolated from the patient's muscle using a Wizard Genomic DNA Purification Kit (Promega cat. A1125) was analyzed by southern blot to determine if deletions were present. After extraction, restriction enzyme digestion was performed with *Pvull and BAMH1*, which have one recognition site in mtDNA resulting in linearization. Electrophoresis was performed on the patient's sample (P) in the presence of a DNA marker (M), three negative controls (CN1-3), a positive control with a single deletion (CP), and undigested, circular mtDNA control (UC). It was transferred to a positively charged nylon membrane for analysis by the southern blot procedure as previously described [15].

### 3.4. Mitochondrial DNA Copy Number Analysis

Multiplex Real-time PCR using a TaqMan assay as previously described [16] was performed on the patient's muscle to determine the copy number of mitochondrial DNA. Briefly, segments of the 12s rRNA gene in mtDNA and the ribonuclease P gene in nuclear DNA (nDNA) were amplified simultaneously, and fluorescently labeled probes were utilized to determine the relative concentrations. Amplification was carried out on a 7500 Fast Real-Time PCR System (Applied Biosystems, Waltham, MA), and the resulting data were analyzed using 7500 software v2.0.6. The level of mtDNA content was calculated using the difference between the threshold cycles (C_t_) of mtDNA and nDNA using the formula 2 × 2^^ΔCt^, where ΔC_t_ is the difference of C_t_ values between the ribonuclease P gene and the mitochondrial 12s rRNA gene. In each run, standard curves were generated for mtDNA and nDNA to verify the linearity and equal amplification efficiency of the two amplicons during polymerase chain reaction.

## 4. Results

**Figure 1:**
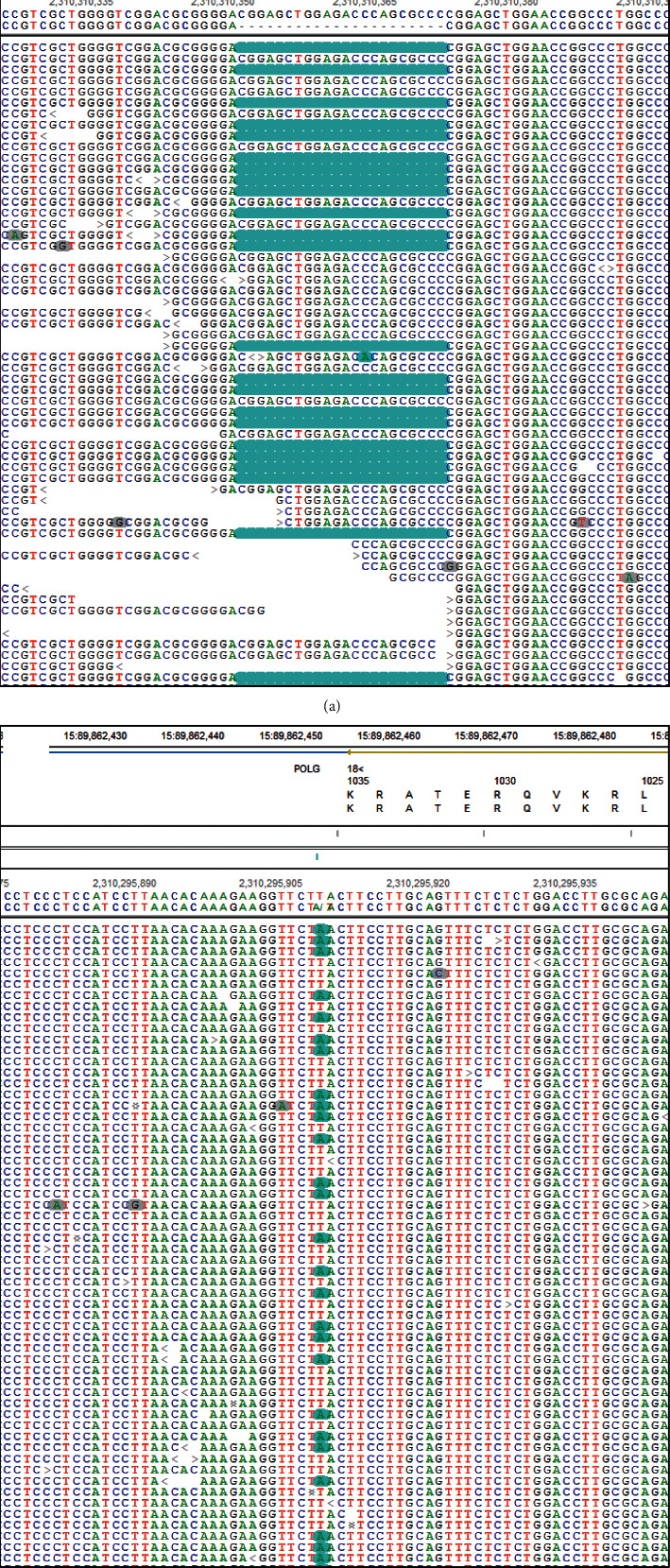
Whole exome sequencing demonstrates compound heterozygous variants in POLG. Pile-ups of sequencing reads show (a) a heterozygous deletion of 22 base pairs in exon 2, c.67_88del, resulting in p.(Gly23Serfs^*∗*^236) and (b) a heterozygous point mutation in intron 19, c.3104 + 3A > T, resulting in skipping of exon 19. The variants are highlighted in blue.

Whole-exome sequencing identified a novel frame shift variant in exon 2 of *POLG*, p.(Gly23Serfs^*∗*^236) (c.67_88del), which existed in compound heterozygosity with a previously reported splice-site mutation in intron 19 of *POLG*, c.3104+3A > T (Figure 1). Based on ACMG criteria, c.3104+3A > T is classified as likely pathogenic with the following criteria: PS3 (well-established functional studies show a deleterious effect [12, 13]), PM2 (absent from population databases), PM3 (detected in trans with a pathogenic variant), PP5 (reputable source reports as pathogenic), and BP4 (computational evidence suggests no impact on gene product) [17]. p.(Gly23Serfs^*∗*^236) is classified as pathogenic based on the following criteria: PVS1 (null variant in a gene where loss of function is a mechanism of disease), PM2 (absent from population databases), PP3 (multiple lines of computational evidence support a deleterious effect), and PP5 (reputable source reports as pathogenic). No additional relevant variants were identified on whole exome sequencing, including in other genes associated with PEO (*POLG2*, *OPA*, *ANT1*, *TWNK*, *RRM2B*, *DNA2*, *TYMP*, *DGUOK*, *TK2*, *MGM1*, and *RNASEH1*). Sanger sequencing was performed on DNA extracted from the patient's muscle and a buccal swab from the patient's child. It confirmed the presence both *POLG* variants in our patient and identified only the c.67_88del variant in the patient's offspring, confirming the variants identified in our patient are in trans (compound heterozygous).

**Figure 2:**
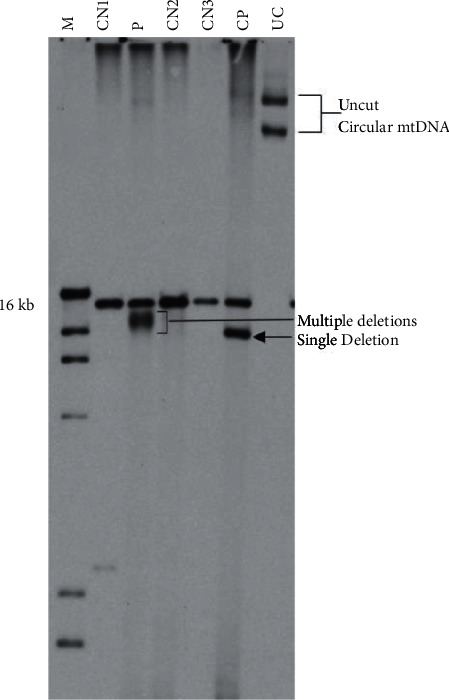
Southern blot for mtDNA deletion analysis. The patient's sample (P) shows an additional band with indistinct margins localizing to a smaller size than the 16 kb expected for intact mtDNA. The indistinct edges of the band represent multiple deletions (compared to lane CP with a single deletion). Patient, P; DNA marker, M; negative controls, CN1-3; positive control with a single deletion, CP; and uncut circular mtDNA control, UC.

Sequencing of mtDNA for common pathogenic point mutations was negative. Southern blot performed on DNA from the patient's muscle showed multiple mtDNA deletions (Figure 2), consistent with the expected pattern in pathogenic *POLG* mutations. mtDNA copy number analysis showed only 48% of the expected value when compared to simultaneously run controls, consistent with mtDNA depletion.

## 5. Discussion

PEO can present at any age with symptoms of bilateral ptosis and ophthalmoparesis progressing in severity over time. Inheritance occurs in both autosomal dominant and recessive fashion, and a variety of genes have been implicated in the development of PEO [7]. *POLG* is the most common gene associated with autosomal dominant PEO, and variants in *POLG*, as seen in our patient, are associated with complex and severe phenotypes [11]. With one exception, all dominant mutations in POLG are point mutations in the polymerase catalytic domain [9, 18]. Other genes implicated in autosomal dominant PEO include *POLG2*, *OPA1*, *ANT1*, *C10orf2*, *RRM2B*, and *DNA2*, which are all nuclear genes involved in maintenance and replication of mtDNA. Autosomal dominant variants in these genes lead to the development of multiple mtDNA deletions [19]. However, mtDNA depletion is not a feature of autosomal dominant conditions, as adequate amounts of all wild-type proteins are present to synthesize an appropriate amount of mtDNA [2, 11]. Autosomal recessive PEO is associated with nonfunctional variants in *POLG*, as well as in *TYMP*, *DGUOK*, *TK2*, *MGM1*, and *RNASEH1* [19]. In recessive states, there may be complete functional absence of one of these enzymes, so in addition to mtDNA deletions, recessive variants can lead to instability and depletion of mtDNA [3, 12].

Our patient harbored a novel *POLG* variant, p.(Gly23Serfs^*∗*^236), occurring in compound heterozygosity with a previously reported pathogenic splice-site variant, c.3104+3A > T. As the novel p.(Gly23Serfs^*∗*^236) variant causes a shift in the reading frame starting in exon 2, the resulting POLG protein is predicted to lose its normal functional domains, including the polymerase and exonuclease domains. Both variants identified in our patient are likely loss-of-function variants because mtDNA depletion was demonstrated in the patient's skeletal muscle, and in addition, neither variant leads to an alteration in the polymerase catalytic domain. Loss-of-function variants in *POLG* are associated with autosomal recessive inheritance, consistent with the compound heterozygosity found in our patient.

The genotype-phenotype correlation in *POLG*-related disorders has not been fully elucidated. Heterogeneous presentations of mitochondrial disease have been previously described in patients harboring the same *POLG* genotype [7, 20, 21]. This suggests the possibility of an oligogenic inheritance with other genes being involved in the disease process. However, we did not identify any additional mutations in genes involved in mitochondrial DNA maintenance. Furthermore, the common overlapping symptoms of PEO in the c.3104+3A > T *POLG* patients (Table 1) argues against other genes being involved in the phenotype seen in our patient. Therefore, the findings of mtDNA depletion, multiple mtDNA deletions, and the resulting phenotypic PEO can reasonably be attributed to the variants discovered in the *POLG* gene.

The c.3104+3A > T variant has been previously reported in compound heterozygosity in four patients (three unrelated families) with phenotypic features consistent with PEO (Table 1) [12, 13]. Previous studies have demonstrated this variant to result in a protein product containing 41 fewer amino acids than the wild-type POLG protein, consistent with skipping of exon 19 [13]. This results in loss of a portion of the finger subdomain, responsible for DNA binding, within the polymerase catalytic domain [12]. Therefore, skipping of exon 19 leads to decreases in both DNA binding affinity and polymerase catalytic activity [13]. Roos et al. [12] previously demonstrated that POLG proteins lacking exon 19 were not capable of synthesizing double-stranded DNA.

If the exon skipping was complete in the c.3104+3A > T variant and if no functional protein was produced by the p.(Gly23Serfs^*∗*^236) variant as expected, our patient would not produce functional mtDNA polymerase and this genotype would be incompatible with life. However, through sequencing of mRNA transcripts, Roos et al. [12] identified a small amount of wild-type *POLG*, and they determined that exon skipping due to the c.3104+3A > T variant is incomplete. They further demonstrated that low levels of the wild-type protein allowed for efficient DNA synthesis of the leading strand, but there was a significant delay in the synthesis of the lagging strand. This leads to DNA products remaining single stranded for prolonged periods, increasing susceptibility to mtDNA deletions [12]. The exonuclease activity of the functional protein is apparently intact, as no increase in point mutations was detected in mtDNA extracted from the patient's muscle [22].

In summary, we present a patient with adult-onset PEO with a novel *POLG* variant, p.(Gly23Serfs^*∗*^236), occurring in compound heterozygosity with a previously reported splice-site variant, c.3104+3A > T.

## Figures and Tables

**Table 1:** Summary of reported patients with the c.3104+3A > T *POLG* variant in compound heterozygosity.

Authors	Sex	Ptosis onset (age, years)	Additional *POLG* variant	Symptoms
Milone et al. [13]	M	62	p.(Phe749Ser)	(i) Moderate bilateral ptosis (ii) Severe ophthalmoplegia (iii) Mild generalized weakness (iv) Distal superficial sensory loss

Milone et al. [13]	M	Mid-40s	p.(Gly848Ser)	(i) Ptosis (ii) Limited eye movements (iii) Exercise intolerance (iv) Sensorineural hearing loss (v) Dysphagia (vi) Generalized weakness

Roos et al.^a^ [12]	M	20	p.(Thr914Pro)	(i) Bilateral ptosis (ii) Ophthalmoplegia (iii) Exercise intolerance (iv) Dysphagia (v) Respiratory weakness

Roos et al.^a^ [12]	M	50	p.(Thr914Pro)	(i) Ophthalmoplegia (ii) Ptosis (iii) Proximal limb weakness (iv) Facial weakness (v) Respiratory weakness

Kurtz, et al. (current)	F	52	p.(Gly23Serfs^*∗*^236)	(i) Ophthalmoplegia (ii) Bilateral ptosis (iii) Exercise intolerance (iv) Generalized muscle weakness (v) Left-sided intention tremor

^a^Siblings.
